# Effects of full-body mirror exposure on eating pathology, body image and emotional states: Comparison between positive and negative verbalization

**DOI:** 10.1371/journal.pone.0257303

**Published:** 2021-09-13

**Authors:** Julia A. Tanck, Andrea S. Hartmann, Jennifer Svaldi, Silja Vocks

**Affiliations:** 1 Department of Clinical Psychology and Psychotherapy, Institute of Psychology, Osnabrück University, Osnabrück, Germany; 2 Department of Clinical Psychology and Psychotherapy, Institute of Psychology, Tübingen University, Tübingen, Germany; University of Pittsburgh, UNITED STATES

## Abstract

Mirror exposure (ME) is an effective technique to improve body image. However, evidence on the underlying mechanisms and the optimal verbalization instruction during ME is lacking. Therefore, this experimental study analyzed mechanisms of ME and therapeutic outcomes by comparing positive (PV) and negative (NV) full-body verbalization. *N* = 73 healthy females were randomized to a PV or an NV condition. PV participants verbalized positively while NV participants verbalized negatively about their whole body. Each participant underwent three standardized ME sessions. Before and after each ME session, positive affect, negative affect and body satisfaction were assessed. Before the first and after the third ME, participants completed questionnaires on cognitive-affective and behavioral aspects of body image, eating pathology and self-esteem. Regarding within-ME changes, the results indicate that positive affect and body satisfaction decreased while negative affect increased in the NV group but not in the PV group. In contrast, regarding between-ME changes, decreased negative affect as well as positive affect and increased body satisfaction were observed in both groups. However, eating pathology remained stable, whereas body-checking behavior increased and the PV condition was followed by higher levels of self-esteem compared to the NV condition. These findings suggest that both PV and NV improve negative affect and body satisfaction between-ME, and thus seem to be effective ME instructions. Given that NV led to increased negative affect within-ME and did not influence self-esteem, PV might represent the favorable instruction during ME for body-satisfied women.

## Introduction

‘Body image’ is broadly defined as the mental representation of the shape, form and size of one’s body [1]. A negative body image can be divided into a perceptual, a cognitive-affective and a behavioral component [2]. The perceptual component encompasses the individual’s subjective view of the dimensions of his/her own body [3, 4], while the cognitive-affective component manifests in body-related thoughts and emotions, e.g., body dissatisfaction [5]. Behavioral aspects of a disturbed body image are evident in body-checking behavior, e.g. feeling for protruding bones or measuring the size of multiple body parts [6, 7], and body avoidance behavior [8, 9]. Body avoidance behavior strategies manifest in the avoidance of seeing one’s body in the mirror [8, 10].

Several therapeutic interventions aim at improving dysfunctional aspects of body image in patients with eating disorders (ED) [11–13]. Mirror exposure (ME) has been shown to be effective for improving behavioral and cognitive-affective aspects of a negative body image [14–17], and is therefore integrated in various cognitive-behavioral therapy manuals for ED [13, 18]. By definition, the primary objective of ME is to guide the patient or participant to systematically describe one’s own body when viewing it in a mirror [11].

However, the implementation of ME as a cognitive-behavioral intervention can take different forms, i.e. it can be described as ‘pure’ [19] or ‘guided’ [19–21]. During ‘pure’ ME, participants are asked to attend to the thoughts and feelings that arise while observing one’s body in the mirror without any avoidance [21]. In ‘guided’ ME, by contrast, the therapist instructs the participants to systematically describe their physical appearance in as much detail and as accurately as possible while viewing one’s body in a full-length mirror [11]. Specifically, participants can either be instructed to systematically describe their body in a neutral, non-judgmental manner of verbalization [19], in a positively valenced way [20, 22] or in a negatively valenced way [23]. Hence, ME can vary in terms of the language, i.e. verbalization, that participants are instructed to use for describing their own body. Participants may be instructed to verbalize positive or negative thoughts and emotions that arise while viewing their body in the mirror, or they may be asked to describe the appearance of their body without judgment [11].

Although there is evidence that ME is effective for improving behavioral and cognitive aspects of body image [11], knowledge on the mechanisms by which ME improves ED symptomatology is still lacking. One of the suggested mechanisms of ME is the habituation to the negative affect that is associated with the exposure to one’s own body [24]. The emotional processing model by Foa and Kozak [25] proposed that changes in cognitive-affective responses require (a) an initial psychophysiological activation followed by (b) a psychophysiological decrease within the session, i.e. within habituation, and (c) a psychophysiological decrease to the next session, i.e. between habituation. In line with theoretical assumptions by Foa and Kozak [25], research on habituation processes of ME has shown a reduction of psychophysiological arousal within [19, 24, 26] and between the ME sessions [16]. With the aim of investigating the habituation mechanism, the addition of an emotional focus by instructing a negative verbalization (NV) of body-related thoughts and emotions might foster psychophysiological arousal, followed by a decrease within and between sessions.

Based on these assumptions, in an experimental study [23], healthy participants with body dissatisfaction were asked to either focus their attention exclusively on their eight least-liked body parts (negative ME condition) or on their eight most-liked body parts (positive ME condition), while verbalizing their accompanying thoughts and emotions. While both conditions were equally effective in increasing body satisfaction, the negative ME initially led to increased negative emotions, i.e., shame or anxiety, which subsequently improved after three to four sessions of 30 minutes duration. Furthermore, the negative ME led to larger improvements in the individual rating of the least-liked body parts [23]. Notably, though, participants in this study had to verbalize their negative thoughts and emotions about their eight least-liked body parts only. However, based on the assumptions and predictions of emotional processing theory [25], the psychophysiological activation within the ME and the habituation process may have been enhanced by a stronger activation of a fear structure, i.e. an NV, for all body parts, which has not yet been examined.

Another potential factor that has been proposed as an underlying mechanism for the effectiveness of mirror exposure is the redirection of the attentional focus towards an overall balanced view of one’s body [27]. Specifically, patients with ED preferentially allocate their focus of attention towards negatively valenced body parts, which seems to result in body dissatisfaction and eating psychopathology [28]. In order to redirect the attentional focus to a balanced viewing pattern, patients with ED might therefore benefit from ME by attending longer to their positively valenced body parts due to the instruction to speak positively about one’s own body [20]. The theoretical conceptualization of a redirection of the attentional focus by ME would therefore suggest a positive verbalization (PV) over a focus on negatively valenced body parts, as the patient also trains to focus on positive aspects of her body, which may consequently alter the attentional bias and associated body dissatisfaction [22]. Indeed, in the aforementioned study conducted by Luethcke et al. [20], PV during ME significantly improved participants’ body dissatisfaction in healthy females with body dissatisfaction. Contrary to the underlying theoretical assumption, though, PV during ME did not change participants’ selective viewing pattern. However, study participants were explicitly instructed to focus on and verbalize the body parts they had already rated as positive, rather than PV on all body parts, which may have diminished the attentional redirection.

Taken together, these findings suggest that the underlying cognitive-affective processes of ME provide essential indications with respect to its mechanisms. However, no study to date has investigated a full-body PV compared to a full-body NV during ME in randomized controlled trials. Hence, the present study was conducted to identify the relationship between full-body verbalization (i.e., PV or NV), irrespective of one’s judgment of the specific body part, and the associated effects on body image and eating pathology.

Against this backdrop, we implemented two versions of ME which have not previously been experimentally tested with respect to the effects on body image. We therefore chose non-clinical participants, who were asked to either positively (PV) or negatively (NV) verbalize about their whole body, irrespective of their subjective evaluation of the addressed body parts. After randomization to PV or NV, all participants underwent three standardized ME sessions. Dependent variables included trait-like eating pathology and body image as well as state affect and state body satisfaction. The study aims were as follows: We sought to compare changes in positive and negative emotions and state body satisfaction from pre- to post-ME within and between the ME sessions depending on PV and NV. Furthermore, we wished to analyze the effects of PV or NV during ME on cognitive-affective and behavioral aspects of body image, eating pathology and self-esteem before the first ME session and after the third ME session. Finally, we analyzed effects of PV and NV on changes in participants’ rating of their most-liked and least-liked body part after the third ME session. Based on previous research, we proposed the following hypotheses: First, we assumed that NV compared to PV would result in decreased positive affect within the ME sessions but that PV would lead to increased positive affect between the ME sessions, because participants were instructed to focus on positively valenced aspects of each body part [20, 22]. In addition, with regard to negative affect, we hypothesized that NV would result in significantly higher negative affect within ME sessions and lower negative affect between ME sessions. As our second hypothesis, we stated that in line with the emotional processing model by Foa and Kozak [25], compared to PV, NV would lead to significantly greater initial arousal, followed by decreases in psychophysiological arousal and thereby to improvements in body satisfaction within and between ME sessions. Third, we expected that both NV and PV would improve eating pathology, cognitive-affective and behavioral aspects of body image and self-esteem, because ME as a cognitive-behavioral intervention appeared to positively influence eating pathology and body image [11]. Fourth, in line with the results of Jansen et al. [23], we hypothesized that the NV would result in greater improvements in participants’ satisfaction with their least-liked body part, whereas the PV would reveal increased satisfaction with the participants’ most-liked body part.

## Material and methods

### Sample and recruitment

All participants provided written consent and received either 25 Euros or study credit as an incentive. The present study was approved by the ethics committee of Osnabrück University (4/71043.5). The sample consisted of non-clinical female participants who were recruited through press releases of the university, leaflets in sports clubs and gyms, social media ads as well as personal contacts. After an initial email contact, the potential participants underwent a structured telephone interview performed by psychology graduate students and supervised by a certified clinical psychologist to assess predefined exclusion criteria, i.e., suicidality, self-harm behavior, current pregnancy, illegal substance abuse or a diagnosed mental disorder (see S1 File for the structure of the standardized telephone screening). All participants completed pre-questionnaires on body image issues and body dissatisfaction, but the selection for inclusion in the present study was not based on these pre-values, as we aimed to recruit a community-based sample with the full range of body dissatisfaction levels usually presented in such samples. Inclusion criteria were age between 18 and 45 years and fluent German-language skills.

**Fig 1:**
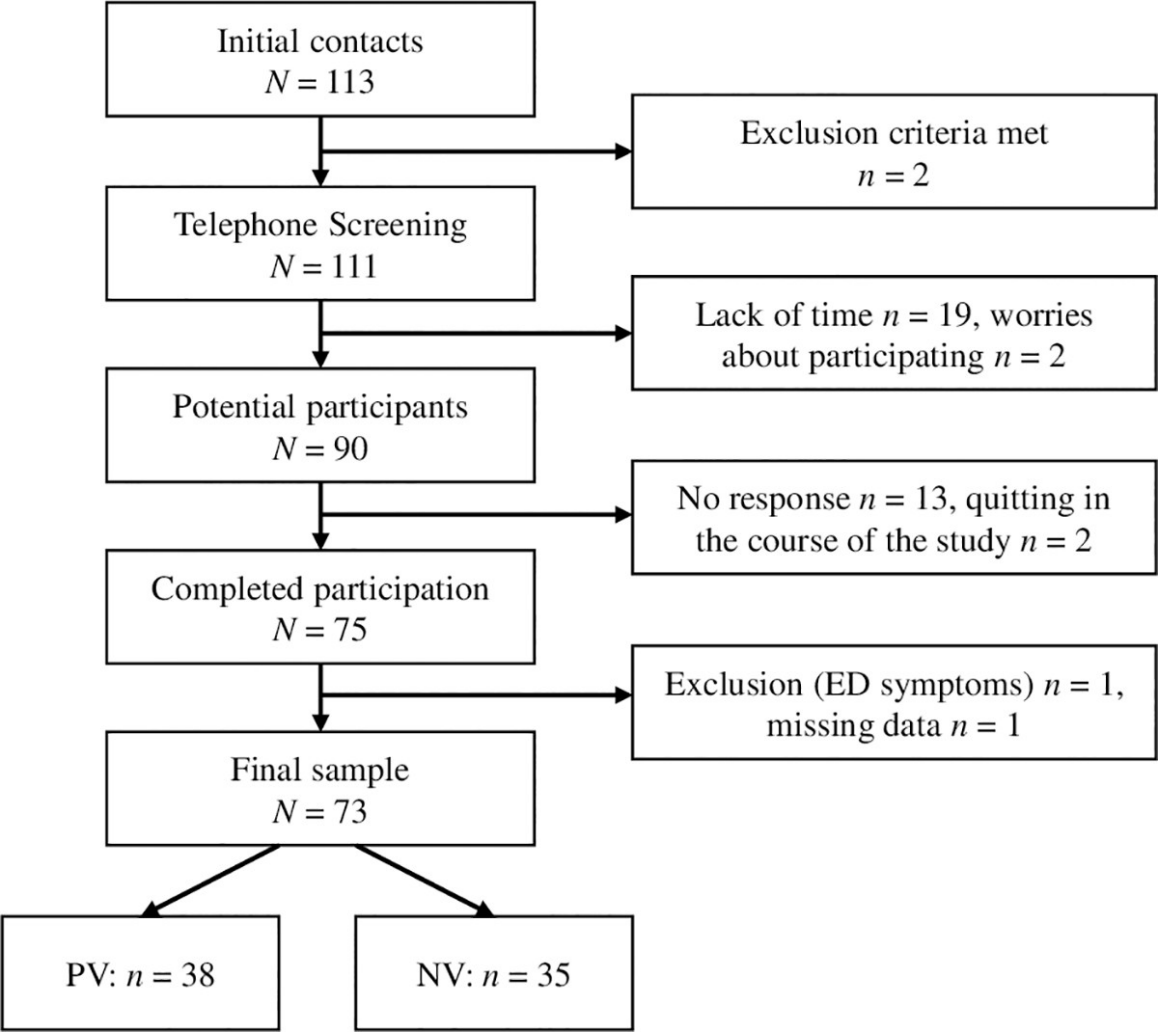
Participant flow chart. PV = Positive Verbalization, NV = Negative Verbalization.

Once participants had passed the screening, a date and time for the first personal appointment at the laboratory of the university was arranged. Out of 113 initial email contacts, 73 participants completed the whole experimental study (*N* = 73). Fig 1 illustrates the full participant flow chart.

### Measures

#### Eating Disorder Examination-Questionnaire (EDE-Q [29]; German-language version [30])

To assess eating pathology concerning symptomatology in AN and BN, we calculated the mean score of the four EDE-Q subscales Restraint, Eating Concern, Shape Concern and Weight Concern. The EDE-Q consists of 22 items which are rated on a Likert scale from 0 (*no days/none of the times/not at all*) to 6 (*every day/every time/markedly*). High scores indicate high levels of eating disorder pathology. Internal consistency in the present study was excellent, i.e., α = .92 for the EDE-Q global score, α = 86 for Shape Concern, α = .76 for Eating Concern, α = .76 for Weight Concern and α = .81 for Restraint.

#### Eating Disorder Inventory-2 (EDI-2 [31]; German-language version [32])

To measure cognitive-affective correlates of body image, i.e., body dissatisfaction or fear of weight gain, typically represented in individuals with EDs such as AN and BN, we included the subscales Body Dissatisfaction and Drive for Thinness from the EDI-2. The subscale Body Dissatisfaction comprises nine items and the subscale Drive for Thinness seven items, rated on a Likert scale from 0 (*never*) to 6 (*always*). High values on both subscales indicate a high expression of dysfunctional aspects of cognitive-affective body image. In the present sample, Cronbach’s α was α = .84 for Body Dissatisfaction and α = .88 for Drive for Thinness.

#### Body Image Avoidance Questionnaire (BIAQ [33]; German-language version [34])

In order to assess behavioral correlates of body image, i.e., body avoidance behavior, we used the BIAQ, which is a self-report measure encompassing 19 items on the four subscales Clothing, Social Activities, Eating Restraint, and Grooming/Weighing. Items are rated on a five-point Likert scale from 0 (*never*) to 4 (*always*). The higher the mean BIAQ score, the higher the body-related avoidance behavior. The internal consistency in the current sample lay at α = .50 for the global BIAQ score.

#### Body Checking Questionnaire (BCQ [35]; German-language version [36])

To measure body-checking behavior as a behavioral correlate of body image, we administered the BCQ, which contains 23 items rated on a five-point Likert scale from 0 (*never*) to 4 (*always*). High BCQ values indicate frequent body-checking behavior. The internal consistency in the present sample was excellent, lying at α = .86.

#### German Single-Item Self-Esteem Scale (G-SISE [37]; German-language version [38])

The single-item G-SISE was used to assess global self-esteem. In contrast to the original scale, we used a four-point Likert scale, as is the case in the ten-item Rosenberg Self-Esteem Scale [37], ranging from 1 (*not at all*) to 4 (*completely*). The G-SISE correlates highly with the ten-item Rosenberg Self-Esteem Scale (*r* = .72 –.80) [37] and can therefore be seen as a reliable and economical instrument to assess self-esteem [38]. The higher the G-SISE score, the higher the global self-esteem.

#### Body Areas Rating Scale (BARS; self-constructed)

To evaluate the satisfaction with the nine body areas focused on during ME, the BARS was implemented. This scale comprises nine items rated on a 7-point Likert scale ranging from 1 (*very dissatisfied*) to 7 (*very satisfied*). Hence, high scores on the BARS indicate high body satisfaction. In addition, the nine body parts had to be ranked from 1 (*least attractive*) to 9 (*most attractive*). The most-liked and least-liked body part was identified individually for each participant in accordance with the ranking between 1 (*least attractive*) and 9 (*most attractive*). In the present study, Cronbach’s α amounted to α = .67 for the rating of the nine body parts.

#### Positive and Negative Affect Schedule–Expanded form (PANAS-X [39]; German-language version [40])

The Negative Affect subscale (10 items) of the PANAS-X was used as a measure of affective states referring to body-related concerns, i.e., feelings of disgust, shame or guilt [7], while the Positive Affect subscale (10 items) was included in order to also cover positive affective states resulting from the positive ME. All items are rated on a five-point Likert scale from 1 (*not at all*) to 5 (*extremely*). High values on both subscales relate to high emotional activation. In the present study, for the six points of measurement, internal consistencies ranged from α = .69 to α = .78 for Negative Affect and from α = .86 to α = .90 for Positive Affect.

#### Body Image States Scale (BISS [41]; German-language version [42])

The BISS was used to assess changes in state body satisfaction as part of the cognitive-affective component of body image. The scale contains six items to evaluate the current satisfaction with various aspects of physical appearance. The higher the mean score of the six items, the higher the body satisfaction. All items were rated on a nine-point Likert scale from 1 (*extremely dissatisfied*) to 9 (*extremely satisfied*). The internal consistencies in the present sample ranged between α = .89 and α = .91.

### Materials

#### Audio files

In order to conduct mirror exposure in a standardized manner, audio files with instructions to describe one’s body were recorded prior to the experiment. We divided the whole body into the following nine body areas: (1) face/teeth/ears/hair, (2) neck/décolleté, (3) breasts, (4) upper arms/elbows, (5) lower arms/hands, (6) stomach/waist/hips, (7) upper back/lower back/bottom, (8) upper legs/knees, (9) lower legs/feet. These body areas were addressed for three minutes each and presented in a standardized order, starting with the (1) face/teeth/ears/hair, and ending with (9) lower legs/feet. Therefore, every ME session included the confrontation with all body parts, i.e., full-body ME. Two audio files were recorded, lasting for 47 minutes each, and utilizing PV and NV as ME instructions. The instructions included identical content apart from a different valence: The PV condition required participants to verbalize exclusively what they liked about their body while the NV required them to verbalize what they disliked. Example instructions for PV and NV can be found in the S1 File.

#### Mirror exposure equipment

The mirror exposure was carried out inside a three-winged mirror cabin which was constructed for the purpose of the study, with a height of 2.12 m and a width of 0.92 m for each wing. This enabled the participants to view their bodies from all angles in line with the respective instruction.

### Procedure

**Fig 2:**
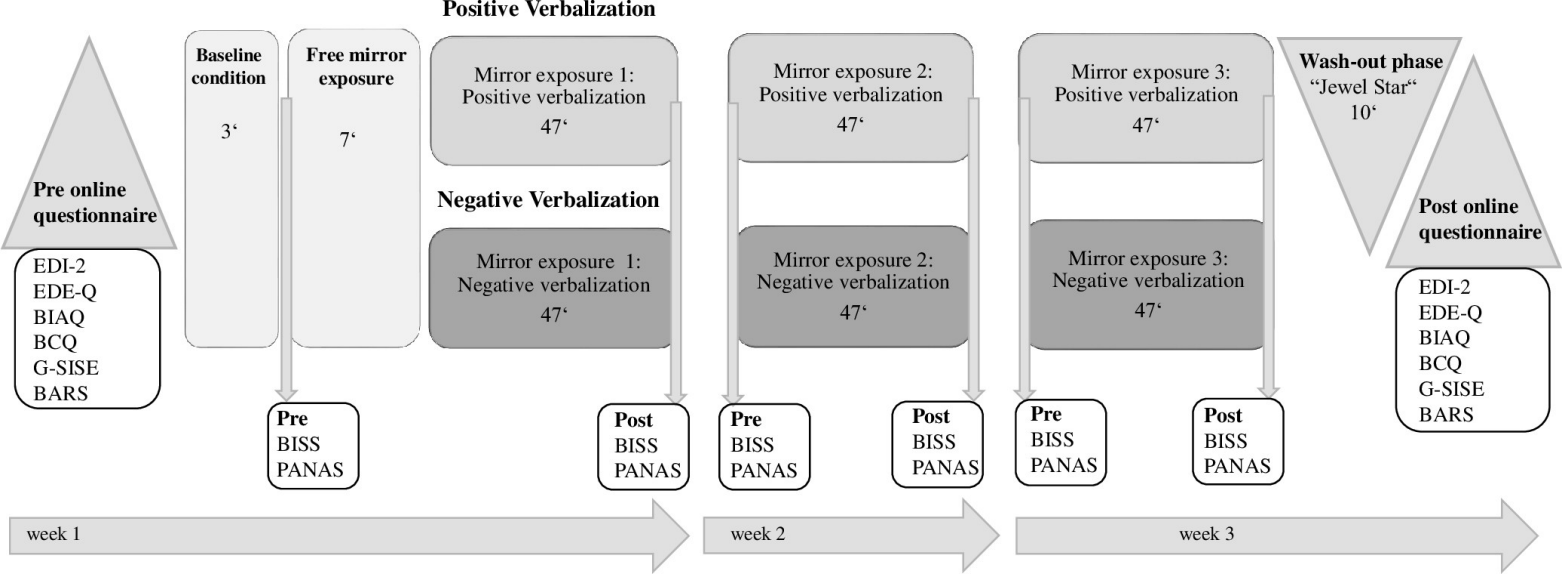
Procedure of the present study. EDI-2 = Eating Disorder Inventory-2; EDE-Q = Eating Disorder Examination Questionnaire; BIAQ = Body Image Avoidance Questionnaire; BCQ = Body Checking Questionnaire; G-SISE = German Single-Item Self-Esteem; BARS = Body Areas Rating Scale; BISS = Body Image States Scale. PANAS = Positive and Negative Affect Schedule.

The data collection took place in two laboratories of Osnabrück University. The experimental study was divided into three ME sessions, including two online questionnaire batteries (pre and post) programmed using Unipark to assess pre- and post-levels of eating pathology, body image and self-esteem. The completion of each online questionnaire battery took about 30–45 minutes. The three ME sessions were implemented with a time interval of three to nine days between the sessions. Before the first ME session, participants were assigned to one of the ME conditions via block randomization. During data collection, a female investigator was present. For protection of privacy, the female investigator sat on a chair in the laboratory and could not see the participant in her underwear at any time. Fig 2 illustrates the procedure of the study.

At the beginning of the first ME session, participants received written and verbal information about the background of the study and provided written consent. The participants were informed that the study was about comparing two variants of ME. After signing the consent form, participants completed the pre-online questionnaire battery on a computer in the laboratory. Subsequently (not relevant for this paper and reported elsewhere), all participants underwent a baseline condition in which they had to describe a collage of nine neutrally validated pictures of the OASIS database [43] for three minutes followed by a seven-minute free-ME with the instruction to verbalize their thoughts and emotions.

Prior to the free-ME, pre-BISS and pre-PANAS were administered as paper-and-pencil versions. Participants then got undressed down to their underwear, put on a bathrobe and were weighed by the investigator before moving inside the mirror cabin and taking off the bathrobe. Directly after completing the 47-minute ME, participants put on the bathrobe and completed the post-BISS and post-PANAS. Finally, participants changed back into their clothes and were offered a short debriefing, in which they were able to talk about activated thoughts and emotions after the ME session. The implementation of ME was identical in all three ME sessions.

After the third ME session, participants played the game “Jewel Legend” for ten minutes on a smartphone. This served as the wash-out phase, through which we aimed to neutralize the activated body-related thoughts and emotions in order to obtain valid data in the post-online questionnaire battery. We chose this particular game because it did not contain any depiction of human bodies or food-related stimuli. The object of the game was to match jewels by their color.

The three laboratory assessments lasted for a total of approximately five hours (i.e., first session two hours, second session one hour, third session two hours).

### Data analysis

Statistical analyses were conducted using IBM SPSS Statistics Version 26. To examine whether PV and NV differentially influence cognitive-affective components of state body image (i.e., BISS, PANAS), we ran three 2 × 2 × 3 mixed-design repeated measures ANOVAs with the between-subjects factor Condition (PV, NV), the within-subjects factors Time (Pre, Post) and Session (S1, S2, S3), and the mean scores of PANAS Positive Affect, PANAS Negative Affect and BISS as dependent variables. To identify the effects of PV vs. NV on eating pathology and body image measured by the EDE-Q, EDI-2, BIAQ, BCQ and G-SISE, we ran two 2 × 2 mixed-design repeated measures MANOVAs with the between-subjects factor Condition (PV, NV) and the within-subjects factor Time (pre, post). We merged the MANOVA groups in line with conceptual associations between the constructs. The first MANOVA included the subscales of the dependent variables EDE-Q and EDI-2 as measures of eating pathology; the second MANOVA included the mean BIAQ and mean BCQ scores as aspects of the behavioral component of body image. To identify the effect of PV and NV on self-esteem, we calculated a 2 × 2 repeated measures mixed ANOVA with the between-subjects factor Condition (PV, NV) and the within-subjects factor Time (pre, post) and G-SISE as the dependent variable. For analyzing effects of PV and NV on the participants’ rating of least-liked and most-liked body parts, we ran a 2 × 2 repeated measures mixed ANOVA, including the between-subjects factor Condition (PV, NV), the within-subjects factor Time (pre, post) and the participants’ rating of the least-liked or most-liked body part as the dependent variable. To follow up significant interaction effects, Bonferroni-adjusted post-hoc *t*-tests with pairwise comparisons were conducted in order to correct for multiple comparisons, for which we applied the Greenhouse-Geisser correction by default to adjust for the lack of sphericity. Partial eta-squared, Hedges’ g_av_ (within-group effects) and *g*_*s*_ (between-group effects) were used as measures of effect size for group differences with defined effect sizes for partial η^2^ (η_p_^2^) as η_p_^2^ = 0.01 (small), η_p_^2^ = 0.09 (medium) and η_p_^2^ = 0.25 (large), and Hedges’ g_av_ and *g*_*s*_ with *g*_*av*,*s*_ = 0.2 (small), *g*_*av*,*s*_ = 0.5 (medium) and *g*_*av*,*s*_ = 0.8 (large) [44].

## Results

### Sample characteristics

The sample consisted of *N* = 73 female participants. The PV participants were on average *M* = 23.63 (*SD* = 3.83) years old, had a mean BMI of *M* = 21.31 kg/m^2^ (*SD* = 2.06) and exercised for *M* = 4.47 hours per week (*SD* = 2.31). Concerning the NV condition, the participants were on average *M* = 22.49 (*SD* = 2.22) years old, had a mean BMI of *M* = 21.34 kg/m^2^ (*SD* = 3.61) and exercised for *M* = 4.71 hours/week (*SD* = 2.51). Prior to the experiment, the groups did not differ in terms of age (*F*(72) = 2.39, *p* = .126), BMI (*F*(70) = .00, *p* = .968) or weekly exercise (*F*(72) = .18, *p* = .673). In addition, the groups did not differ in pre-values of the EDE-Q global score (*F*(72) = 3.23, *p* = .076) as well as its subscales Restraint (*F*(72) = .52, *p* = .475) and Weight Concern (*F*(72) = 1.71, *p* = .195). However, the groups displayed significant differences in the pre-values with respect to the EDE-Q subscales Eating Concern (*F*(72) = 4.33, *p* = .041) and Shape Concern (*F*(72) = 4.52, *p* = .037), with significantly higher scores in the NV condition. The groups did not differ in their EDI-2 scores (Body Dissatisfaction [*F*(72) = .54, *p* = .464]; Drive for Thinness [*F*(72) = 1.52, *p* = .222]), BIAQ (*F*(72) = .60, *p* = .441), BCQ (*F*(72) = .51, *p* = .476), G-SISE (*F*(72) = 1.00, *p* = .320) and BARS ([BARS global *F*(72) = 3.48, *p* = .066]; BARS least-liked body part [*F*(72) = 1.67, *p* = .201]; BARS most-liked body part [*F*(72) = .03, *p* = .874]). Means and standard deviations of all pre-ME and post-ME measures are shown in Table 1.

**Table 1:** Means and standard deviations for eating pathology, body image, self-esteem and satisfaction with body parts.

	Positive Verbalization (*n* = 38)				Negative Verbalization (*n* = 35)			
	*M*	*SD*	*Min*	*Max*	*M*	*SD*	*Min*	*Max*
*Eating Disorder Examination Questionnaire (EDE-Q)*								
Restraint								
pre-ME	0.96	0.99	0.00	3.40	1.13	1.07	0.00	3.60
post-ME	0.83	0.95	0.00	3.80	1.23	1.08	0.00	4.00
Eating Concern								
pre-ME	0.33	0.47	0.00	2.00	0.61	0.69	0.00	2.60
post-ME	0.33	0.52	0.00	2.00	0.57	0.70	0.00	3.00
Weight Concern								
pre-ME	0.89	0.81	0.00	3.20	1.18	1.06	0.00	4.20
post-ME	0.95	0.81	0.00	3.80	1.41	1.17	0.00	4.60
Shape Concern								
pre-ME	1.20	0.70	0.00	3.25	1.67	1.16	0.00	4.00
post-ME	1.28	0.89	0.25	3.75	1.86	1.17	0.25	4.88
EDE-Q global score								
pre-ME	0.84	0.61	0.00	2.49	1.15	0.83	0.06	3.33
post-ME	0.85	0.70	0.06	2.74	1.27	0.90	0.09	3.37
*Eating Disorder Inventory 2 (EDI-2)*								
Body Dissatisfaction								
pre-ME	3.16	0.41	1.89	3.78	3.09	0.35	2.22	3.78
post-ME	3.16	0.33	2.56	3.78	3.05	0.36	2.33	3.89
Drive for Thinness								
pre-ME	2.36	0.86	1.14	5.00	2.62	1.00	1.14	4.71
post-ME	2.35	0.96	1.14	5.00	2.58	1.10	1.29	5.29
*Body Image Avoidance Questionnaire (BIAQ)*								
pre-ME	0.96	0.20	0.63	1.47	1.00	0.32	0.42	1.53
post-ME	1.02	0.32	0.53	1.47	1.03	0.40	0.42	2.11
*Body Checking Questionnaire (BCQ)*								
pre-ME	0.80	0.35	0.26	1.39	0.87	0.44	0.17	2.00
post-ME	1.86	0.40	1.17	2.87	2.01	0.60	1.13	3.65
*German Single-Item Self-Esteem*								
pre-ME	3.00	0.62	2	4	2.86	0.60	2	4
post-ME	3.13	0.74	1	4	2.74	0.66	2	4
*Body Areas Rating Scale (BARS)*								
BARS global score								
pre-ME	4.99	0.67	3.44	6.44	4.65	0.84	3.00	6.11
post-ME	5.06	0.84	3.44	6.89	4.47	1.02	2.56	6.33
Least-liked body part								
pre-ME	3.24	1.94	1.00	7.00	2.71	1.47	1.00	7.00
post-ME	3.58	1.95	1.00	7.00	2.80	1.62	1.00	7.00
Most-liked body part								
pre-ME	6.16	1.24	1.00	7.00	6.11	1.16	1.00	7.00
post-ME	6.05	1.39	1.00	7.00	6.04	1.26	3.00	7.00

*Note*. *M* = Mean; *SD* = standard deviation; ME = Mirror exposure; *EDE-Q* Minimum = 0, Maximum = 6; *EDI-2* Minimum = 0, Maximum = 6; *BIAQ* Minimum = 0, Maximum = 4; *BCQ* Minimum = 0, Maximum = 4; *G-SISE* Minimum = 1, Maximum = 4; *BARS* Minimum = 0, Maximum = 7.

### Condition-specific effects of mirror exposure on positive affect and negative affect

#### Positive affect

The 2 × 2 × 3 repeated measures mixed-design ANOVA yielded no significant main effect of Condition (*F*(1, 70) = 2.01, *p* = .16, η^2^_p_ = .03), but did yield a significant main effect of Time (*F*(1, 70) = 7.58, *p* = .008, η^2^_p_ = .10), qualified by a significant two-way interaction of Time × Condition (*F*(1, 131.21) = 11.19, *p* = .001, η^2^_p_ = .14). Post-hoc Bonferroni-adjusted comparisons revealed that PANAS Positive Affect significantly decreased from pre- to post-ME in the NV (*M*_*diff*_ = .213, *SE* = .050, *p* < .001) but not in the PV (*M*_*diff*_ = —.021, *SE* = .049, *p* = .672), i.e., the PV and the NV significantly differed in terms of changes in positive affect from pre- to post-ME. The inspection of effect sizes, i.e., Hedges’ g, showed from pre-ME to post-ME *g*_*av*_ = .63 within the first ME, *g*_*av*_ = .35 within the second ME and *g*_*av*_ = .15 within the third ME of the NV. The PV showed effect sizes of *g*_*av*_ = .21 within the first ME, *g*_*av*_ = .29 within the second ME and *g*_*av*_ = .01 from pre to post within the third ME.

We further found a significant main effect of Session (*F*(2, 140) = 4.54, *p* = .012, η^2^_p_ = .06), indicating that the mean positive affect decreased between the sessions overall, qualified by a significant two-way interaction of Session × Time (*F*(2, 140) = 7.92, *p* = .003, η^2^_p_ = .10). Post-hoc Bonferroni *t*-test results revealed significant differences from pre-ME to post-ME in the first session (S1: *M*_*diff*_ = .254, *SE* = .057, *p* < .001), whereas the mean positive affect did not change significantly from pre- to post-ME in session two and session three (S2: *M*_*diff*_ = .010, *SE* = .048, *p* = .843; S3: *M*_*diff*_ = .025, *SE* = .053, *p* < .635). The calculation of effect sizes revealed *g*_*av*_ = .38 within the first session, *g*_*av*_ = .01 within the second session and *g*_*av*_ = .04 within the third session. There were no other significant main effects or interactions (all *F*s < 1.88, all *p*s > .13).

#### Negative affect

The 2 × 2 × 3 ANOVA revealed no significant main effect of Time (*F*(1, 69) = 1.51, *p* = .22, η^2^_p_ = .02), but did reveal a significant main effect of Condition (*F*(1, 69) = 5.21, *p* = .026, η^2^_p_ = .07), qualified by a significant two-way interaction of Time × Condition (*F*(1, 132.29) = 8.49, *p* = .005, η^2^_p_ = .11). Post-hoc comparisons revealed that the NV, but not the PV, resulted in significant increases in PANAS Negative Affect from pre- to post-ME (NV: *M*_*diff*_ = .046, *SE* = .037, *p* = .006; PV: *M*_*diff*_ = .046, *SE* = .037, *p* = .221). In terms of effect sizes, we calculated *g*_*av*_ = .32 within the first ME, *g*_*av*_ = .35 within the second ME and *g*_*av*_ = .30 within the third ME of the NV. The PV showed effect sizes of *g*_*av*_ = .48 within the first ME, *g*_*av*_ = .22 within the second ME and *g*_*av*_ = .15 within the third ME.

Furthermore, there was a significant main effect of Session (*F*(1.50, 103.16) = 31.63, *p* < .001 η^2^_p_ = .31), indicating that the mean negative affect decreased between the sessions overall, and a significant interaction of Session × Condition (*F*(1.50, 103.16) = 3.48, *p* = .034, η^2^_p_ = .05). Pairwise comparisons showed that mean negative affect scores were higher within the NV compared to the PV in session one (S1: *M*_*diff*_ = .220, *SE* = .089, *p* = .016), while the negative affect in session two and session three did not differ significantly between the groups (S2: *M*_*diff*_ = .086, *SE* = .055, *p* = .123; S3: *M*_*diff*_ = .105, *SE* = .055, *p* = .054). The calculation of effect sizes revealed *g*_*s*_ = 0.56 for the first session, *g*_*s*_ = 0.37 for the second session and *g*_*s*_ = 0.48 for the third session. There were no other significant main effects or interactions (all Fs < 2.42, all ps > .09). The means and standard deviations of positive affect and negative affect can be found in Table 2.

**Table 2:** Means and standard deviations for positive affect, negative affect and body satisfaction depending on the instructed verbalization.

	Positive Verbalization		Negative Verbalization	
	(*n* = 38)		(*n* = 35)	
Variables	*M*	*SD*	*M*	*SD*
*Positive and Negative Affect Schedule*: *Positive Affect*^*a*^				
Session 1				
pre-ME	2.59	0.62	2.46	0.68
post-ME	2.42	0.72	2.06	0.59
Session 2				
pre-ME	2.18	0.64	2.28	0.57
post-ME	2.37	0.67	2.07	0.54
Session 3				
pre-ME	2.27	0.71	2.17	0.68
post-ME	2.31	0.73	2.09	0.56
*Positive and Negative Affect Schedule*: *Negative Affect*^*b*^				
Session 1				
pre-ME	1.34	0.24	1.46	0.45
post-ME	1.22	0.26	1.55	0.59
Session 2				
pre-ME	1.21	0.19	1.21	0.26
post-ME	1.17	0.21	1.35	0.42
Session 3				
pre-ME	1.12	0.18	1.19	0.23
post-ME	1.15	0.19	1.29	0.39
*Body Image States Scale*: *Body Satisfaction*^*c*^				
Session 1				
pre-ME	6.15	1.13	5.84	1.38
post-ME	5.88	1.44	4.99	1.71
Session 2				
pre-ME	6.21	1.07	6.09	1.26
post-ME	6.20	1.35	5.34	1.80
Session 3				
pre-ME	6.18	1.03	5.90	1.15
post-ME	6.23	1.31	5.81	1.54

*Note*. *M* = Mean; *SD* = standard deviation; ME = Mirror exposure

^a^ Positive Verbalization *n* = 37, Negative Verbalization *n* = 35

^b^ Positive Verbalization *n* = 38, Negative Verbalization *n* = 33

^c^ Positive Verbalization *n* = 34, Negative Verbalization *n* = 33.

### Condition-specific effects of mirror exposure on body satisfaction

The 2 × 2 × 3 repeated measures mixed-design ANOVA with mean BISS scores as dependent variables revealed no significant main effect of Condition (*F*(1, 65) = 3.25, *p* = .08, η^2^_p_ = .05), but did reveal a significant main effect of Time (*F*(1, 65) = 18.24, *p* < .001, η^2^_p_ = .22) and a significant two-way interaction of Time × Condition (*F*(1, 65) = 11.76, *p* = .001, η^2^_p_ = .15). Subsequent Bonferroni-adjusted pairwise comparisons indicated that NV participants showed a significant decrease in terms of their state body satisfaction from pre- to post-ME, while the PV participants showed no significant change in state body satisfaction (NV: *M*_*diff*_ = .704, *SE* = .130, *p* < .001; PV: *M*_*diff*_ = .077, *SE* = .128, *p* = .551). The inspection of effect sizes revealed *g*_*av*_ = 0.56 within the first ME, *g*_*av*_ = 0.50 within the second ME and *g*_*av*_ = 0.37 within the third ME of the NV. The PV showed effect sizes of *g*_*av*_ = 0.23 within the first ME, *g*_*av*_ = 0.03 within the second ME and *g*_*av*_ = 0.04 from pre to post within the third ME.

We found a significant main effect of Session (*F*(2, 130) = 5.39, *p* = .007, η^2^_p_ = .14) indicating that the mean body satisfaction increased between the sessions overall. In addition, there was a significant two-way interaction of Session × Time (*F*(2, 130) = 4.79, *p* = .006, η^2^_p_ = .15). Post-hoc comparisons revealed that body satisfaction decreased from pre-ME to post-ME within all sessions (S1: *M*_*diff*_ = .557, *SE* = .116, *p* < .001; S2: *M*_*diff*_ = .381, *SE* = .113, *p* = .001; S3: *M*_*diff*_ = .233, *SE* = .099, *p* = .022). The calculation of effect sizes revealed *g*_*av*_ = 0.38 for the first session, *g*_*av*_ = 0.27 for the second session and *g*_*av*_ = 0.17 for the third session. The two-way interaction of Session × Condition (*F*(2, 130) = 0.26, *p* = .77, η^2^_p_ = .00) and the three-way interaction Time × Session × Condition (*F*(1.85, 120.39) = 0.39, *p* = .663, η^2^_p_ = .01) did not turn out to be statistically significant. Table 2 contains the means and standard deviations of body satisfaction.

### Overall condition-specific effects of mirror exposure on eating pathology, body image, self-esteem, satisfaction with body parts

The 2 × 2 repeated measures MANOVA on eating pathology (EDE-Q Restraint, EDE-Q Eating Concern, EDE-Q Weight Concern, EDE-Q Shape Concern, EDE-Q global score, EDI-2 Body Dissatisfaction, EDI-2 Drive for Thinness) revealed no significant main effects of either Time (*F*(6, 66) = 1.80, *Wilks’* λ = .86, *p* = .111, η^2^_p_ = .14) or Condition (*F*(6, 66) = 1.78, *Wilks’* λ = .86, *p* = .118, η^2^_p_ = .14) and no significant two-way interaction of Time × Condition (*F*(6, 66) = 1.23, *Wilks’* λ = .90, *p* = .304).

With respect to body image (BIAQ, BCQ), the 2 × 2 repeated measures MANOVA yielded a significant main effect of Time (*F*(2, 70) = 697.44, *p* < .001, *Wilks’* λ = .05, η^2^_p_ = .95) qualified by a significant post-hoc ANOVA of the dependent variable BCQ (*F*(1, 71) = 1173.90, *p* < .001, η^2^_p_ = .94). There was no significant main effect of Condition (*F*(2, 70) = 0.56, *p* = .574, *Wilks’* λ = .98, η^2^_p_ = .02). Additionally, Bonferroni-adjusted post-hoc *t*-tests indicated significant increases in BCQ scores from pre to post after both PV (*M*_*diff*_ = 1.059, *SE* = .044, *p* < .001) and NV (*M*_*diff*_ = 1.140, *SE* = .046, *p* < .001). The calculation of effect sizes revealed *g*_*av*_ = 2.81 for the PV and *g*_*av*_ = 2.19 for the NV.

In terms of self-esteem, the 2 × 2 ANOVA with mean G-SISE scores as dependent variable revealed no significant main effect of Time (*F*(1, 71) = .02, *p* = .876, η^2^_p_ = .00) and no significant main effect of Condition (*F*(1, 71) = 3.42, *p* = .068, η^2^_p_ = .05), but did reveal a significant two-way interaction of Time × Condition (*F*(1, 71) = 4.92, *p* = .030, η^2^_p_ = .07). Bonferroni-adjusted post-hoc *t*-tests indicated differences between G-SISE at post time points (*M*_*diff*_ = .389, *SE* = .165, *p* = .021), i.e., higher G-SISE scores after PV and lower scores after NV, with an effect size of *g*_*av*_ = 0.55.

Analyzing changes in the rating of the least-liked body part, the 2 × 2 ANOVA with mean BARS scores of the least-liked body part as dependent variable revealed no significant main effect of Time (*F*(1, 71) = 1.08, *p* = .302, η^2^_p_ = .02), and no significant main effect of Condition (*F*(1, 71) = 3.29, *p* = .074, η^2^_p_ = .04). The two-way interaction of Time × Condition (*F*(1, 71) = .39, *p* = .535, η^2^_p_ = .00) was not statistically significant. Concerning the effects of PV or NV on the rating of the most-liked body part, the 2 × 2 ANOVA did not reveal a significant main effect of Time (*F*(1, 71) = .26, *p* = .611, η^2^_p_ = .00) or Condition (*F*(1, 71) = .02, *p* = .876, η^2^_p_ = .00) or a significant two-way interaction of Time × Condition (*F*(1, 71) = .00, *p* = .958, η^2^_p_ = .00). All means and standard deviations of measures on eating pathology, body image, self-esteem and satisfaction with body parts can be found in Table 1.

## Discussion

The present experimental study sought to compare the effects of two variations of ME, i.e., PV and NV, on positive and negative affect, cognitive-affective and behavioral aspects of body image (body dissatisfaction, drive for thinness, body checking and avoidance behavior), eating pathology, and self-esteem, as well as potential working mechanisms of ME. In line with our first hypothesis, the NV reduced positive affect within the ME sessions, while the PV did not yield significant changes in positive affect. Hence, it was shown that positive affect within the ME sessions was only reduced when negatively describing one’s body. However, contrary to our hypothesis, mean positive affect decreased between the three ME sessions in both conditions. Thus, the manner of describing one’s body, i.e., PV and NV, does not seem to play a central role in the development of positive affect between the ME sessions. This finding does not correspond to previous research on effects of positive ME [20, 22]. In contrast to Glashouwer et al. [22], we instructed our participants to positively describe all body parts instead of only focusing on previously positively rated body parts. Therefore, the participants in our study were required to positively describe individually negatively valenced body parts, which might have influenced the positive affective state.

Nevertheless, the first ME session seems to have the greatest impact on decreases in positive affect from pre- to post-ME, as sessions two and three no longer influenced levels of positive affect. Perhaps surprisingly, the decreased positive affect in session one occurred in both ME conditions. Thus, ME seems to be an aversive situation even with the instruction to positively verbalize one’s own body. A possible explanation for these findings might be that body image can be divided into negative and positive body image [45]. Positive body image goes beyond the absence of negative body image and fosters the acceptance and appreciation of one’s own body [46]. Possible threats to positive body image include being weighed and conversing about one’s own body [47]. As such, being weighed prior to the first ME session and then speaking about one’s body might have represented a threat to the participants’ positive body image and resulted in lowered positive affect within the first ME session in our study.

In line with our hypothesis, the NV indeed heightened the negative affect within the ME sessions, whereas negative affective states remained stable in PV. Thus, one might conclude that healthy females only seem to develop negative emotions when they are specifically instructed to negatively verbalize their bodies. These findings are in line with research on the time courses of negative affect during exposure therapy [48]. Following the assumptions of Foa and Kozak [25], a prerequisite for successful habituation is the activation of the fear network. As fear was covered within the PANAS Negative Affect subscale, the NV seems to activate the fear structure within ME. However, it remains open whether the activation of the fear network is necessary for achieving greater benefits from ME for patients with ED. Another possible explanation for the discrepant results between PV and NV might be that the NV group displayed significantly higher pre-values of eating pathology measures, i.e., Eating Concern and Shape Concern, which might have led to decreased positive and increased negative affect within ME.

In accordance with our hypothesis, we found decreased mean negative affect between the sessions in both groups. However, concerning differences between the groups, session one differed significantly from session two and session three, as participants in the NV condition showed higher mean negative affect than participants in the PV condition. As is the case with positive emotional activation, for sessions two and three, no significant differences between the groups emerged. As hypothesized, the instructed negative verbalization seems to strongly activate negative affect, especially during the first ME session, and continuously subsides thereafter [23]. Jansen et al. [23] found that negative ME induced negative feelings within sessions one and two, but this was followed by mood improvements starting at the half-way point of session three. Consequently, the initial negative affect induced by negatively verbalizing parts of the body (as described by Jansen et al. [23]) or the whole body (as in the present study) seems to improve during repeated ME sessions. In contrast to Jansen et al. [23], the differences between the groups concerning negative affect seemed to disappear from the second ME session onwards in our study, which might be explained by our ME session length of 47 minutes compared to 30 minutes in the study by Jansen et al. [23].

Our findings are in line with previous research on ME in non-clinical women, which demonstrated that ME was effective concerning the between-session reduction of negative thoughts and feelings of ugliness overall [17, 19]. Within 50 minutes of exposure, the patient is expected to experience a decrease of fear during prolonged exposure, i.e., within habituation [49], along with a decreased peak intensity of fear activation between sessions [48, 49]. Hence, our results provide evidence that the emotional processing theory [25] might be applicable in the context of ME.

Contradictory to our second hypothesis, participants in the NV condition experienced a decrease in body satisfaction after negatively verbalizing about their body. This finding calls the benefits of NV into question, as contrary to our intention, participants felt less satisfied with their bodies within the ME sessions. Furthermore, the PV did not affect body satisfaction within the ME sessions, which contradicts several previous findings of beneficial effects of positive ME on body satisfaction [20, 22]. Since the self-evaluation of body satisfaction was high in the present study, the lack of increase in body satisfaction appears plausible, or alternatively may reflect a ceiling effect. Previous experimental research in subclinical populations included participants who evaluated themselves as being dissatisfied with their body [20, 22]. Therefore, participants with a higher degree of body dissatisfaction might benefit more in terms of increases in state body satisfaction, as found in these previous studies. Between-session changes indicate that body satisfaction increased between session one and session two and slightly decreased between session two and session three. In conclusion, both PV and NV seem to provoke short-term decreases and long-term increases in body satisfaction. In line with our findings on effects of ME concerning changes in body satisfaction in a sample of body-satisfied women, Svaldi et al. [50] investigated the effect of ME on body satisfaction in individuals with high or low baseline levels of body satisfaction. The authors found no significant influence of ME on body satisfaction in individuals with high baseline body satisfaction.

Our third aim was to investigate the therapeutic effects of PV and NV on eating pathology, cognitive-affective and behavioral aspects of body image and self-esteem. In contrast to previous research in non-clinical but body-dissatisfied women [19, 23], both PV and NV did not influence eating pathology, including body dissatisfaction. Our results suggest that improvements in body dissatisfaction and ED symptomatology through ME cannot be transferred to body-satisfied women. In line with a study by Vocks, Legenbauer, Wächter et al. [26], who found a higher vulnerability to changes in body satisfaction the higher the participants’ body image concerns, our finding suggests that participants’ eating pathology should be taken into account when investigating changes in body-related constructs.

Contrary to our hypothesis and previous research [8, 23], both PV and NV triggered higher levels of body-checking behavior after the third ME session compared to before the first ME session. Possible explanations for this might be traced back to the cognitive-behavioral model of EDs [9]. As a consequence, the desire to check one’s body with the aim of regulating aversive emotions and cognitions might have been unintentionally induced [51]. Our results might provide evidence that the behavioral response to ME exercises conducted alone seems to significantly differ from the behavioral response to a therapeutically guided ME, as other experimental studies including therapeutic guidance did not find increased frequency of body-checking behavior as a result of ME [20, 23]. A clinical implication for body image therapy might be that the presence of the therapist seems to play an important role in behavioral body image outcomes.

In terms of self-esteem, as assumed in our hypothesis, the PV participants reported higher levels of self-esteem compared to the NV participants, thus emphasizing the strong link between body image and self-esteem [52, 53]. Our results contribute to the findings of Hoffmeister et al. [54], who reported that self-esteem increased significantly after ME among unrestrained eaters, but not among restrained eaters. As a clinical implication of this, it would appear to be appropriate to address self-esteem at advanced phases of therapy for EDs, i.e., when patients are already able to reflect upon and practice healthy eating behavior [54].

Contradicting our fourth hypothesis, neither NV nor PV led to improved ratings of either least-liked or most-liked body parts. In contrast to the findings of Jansen et al. [23], the NV did not result in increased satisfaction with the least-liked body part in our study. Moreover, the satisfaction with the most-liked body part remained stable as well. However, the descriptive values revealed that the females in our study were relatively satisfied–even with their least-liked body part–before participating in the study (see Table 1). Jansen et al. [23] included body-dissatisfied females and implemented five sessions of ME. Hence, improvements in the rating of disliked body parts or liked body parts seems to be dependent on pre-levels of body dissatisfaction and the frequency of ME sessions. Future studies should therefore investigate effects of PV and NV among patients with ED in order to identify the most promising technique to improve the rating of least-liked and most-liked body parts.

Nonetheless, some limitations should be mentioned when interpreting the results of the present study. Due to the standardized audio-instructed ME, it was not possible to react to the participants’ statements. In addition, pre-recorded instructions leave little room for flexible deepening of focus, which could have undermined the effectiveness of the intervention. However, we opted for audio-tape instructions for two main reasons. First, this ensured the same duration of body exposure for each body part and each ME. Second, we avoided experimenter effects, which may have influenced the emotional activation. Future research should replicate the study with an experimenter being present in order to deepen the emotional focus and to adjust to the therapy context.

A further limitation refers to the instructions to speak about one body part at a time, which was not objectively controlled for. Moreover, the adherence to the instructions was not monitored. Therefore, the participants might not have followed the instructions, and may have focused on body parts other than those expected, or may have expressed thoughts and emotions that were not in accordance with the respective condition, i.e., PV or NV. However, we aimed to standardize the sequence of body parts in order to create equal conditions for PV and NV. Besides, it seems possible that positively verbalizing about disliked body parts and vice versa might either not represent the authentic feeling towards that specific body part or might even not be realizable under audiotaped instructions. To test this, future studies should involve a therapist to individualize the ME instructions. Another limitation concerning the methodology lies in the lack of power analysis prior to the study and the lack of follow-up measurements of emotional states. Previous research on emotional time courses after body checking, as another method of body exposure, emphasized the importance of follow-up assessments, as decreases in emotional arousal were found to occur 15–30 minutes after the body-checking episodes [51]. Given that state alterations of negative affect, positive affect and body satisfaction were only examined once directly after each experimental condition, future research should consider employing several post-treatment measurements to analyze time courses.

Additionally, three ME sessions might not have been sufficient to target beneficial changes in eating pathology and body image concerns, as previous research has suggested that five sessions of ME might be necessary to achieve improvements of body image disturbance [23]. Furthermore, the review by Klimek et al. (2021) revealed large effects of ME on body image concerns at a dosage of more than 120 minutes of ME [17]. Thus, future studies investigating eating pathology and body image should implement a minimum of five ME sessions, a dosage of at least 120 minutes of ME and follow-up intervals to evaluate long-term therapeutic effects. Moreover, as the internal consistency of the BIAQ to assess body-related avoidance behavior was poor, the non-significant effect concerning this aspect of body image might be due to the lack of reliability in the assessment. Finally, given that our sample consisted of body-satisfied women, the effects found in our study might not be transferable to clinical samples or samples with high body dissatisfaction. Therefore, clinical implications should be drawn with caution.

Nevertheless, potential implications on mechanisms regarding ME might be derived from the findings of the present study: A duration of 50 minutes per exposure session seems to be favorable in order to activate habituation processes [17, 49]. Other studies implemented ME sessions lasting between 30 and 40 minutes [19, 23], which might have been too short for exposure treatment and the activation of habituation processes. Concerning the effect of pre-levels of body satisfaction on the emotional response to viewing oneself in the mirror, women with high body satisfaction do not seem to benefit as much as women with low body satisfaction in terms of the reduction of body-related negative thoughts and emotions [50].

## Conclusion

In sum, our study examined positive and negative affect as well as state body satisfaction within and between ME sessions. Within the sessions, PV led to stable emotional activation, whereas NV resulted in worsened emotional states. Between the sessions, however, the groups only differed regarding session one, as the NV participants showed reduced positive affect and increased negative affect, whereas the group differences disappeared in sessions two and three. Concerning between-session habituation, i.e. the overall changes in affect and state body image over the three ME sessions, repeated ME resulted in reduced positive affect and negative affect as well as increased state body satisfaction for both conditions. Thus, both PV and NV worsened positive affect but improved negative affect and body satisfaction between-ME, but NV induced worsened positive and negative affect within-ME. As such, the present findings suggest that PV might be the favorable ME instruction in body-satisfied populations. Furthermore, our findings provide insights into the onset of body image disturbances, based on the high vulnerability to negative emotional processing of one’s own body when a negative body description is instructed. Future studies should investigate the effects of PV and NV among women with EDs in order to ascertain differences concerning emotional activation between non-clinical and clinical samples.

## Supporting information

S1 File (DOCX)

S1 Data (SAV)
