# Supplementary material for: Effects of full-body mirror exposure on eating pathology, body image and emotional states: Comparison between positive and negative verbalization
Source: PLoS One. 2021 Sep 13;16(9):e0257303. doi: 10.1371/journal.pone.0257303 (PMC8437269; doi:10.1371/journal.pone.0257303)
Supplement: S1 File — (DOCX) [file pone.0257303.s001.docx]

**S1 File**

**Example instructions for body part ‘stomach, waist and hips’**

**Example for PV**

*“Now look at your stomach, waist and hips. Concentrate on those parts of your stomach, waist and hips, that you are pleased with or that you like. Describe in as much detail as possible what you like and why.”* [90 seconds for the participant to speak] *“You are still looking at your stomach, waist and hips. Touch your stomach, waist and hips. What feels good? Which textures on your stomach, waist and hips do you like?”* [90 seconds for the participant to speak].

**Example for NV**

*“Now look at your stomach, waist and hips. Concentrate on those parts of your stomach, waist and hips that you are not pleased with or that you dislike. Describe in as much detail as possible what you dislike and why.”* [90 seconds for the participant to speak] *“You are still looking at your stomach, waist and hips. Touch your stomach, waist and hips. What feels not good? Which textures on your stomach, waist and hips do you dislike?”* [90 seconds for the participant to speak]

| **Question** | **Answer** | |
| --- | --- | --- |
| Are you between 18 and 45 years old? | □ yes | □ no |
| Do you speak German fluently? | □ yes | □ no |
| Do you consume drugs or alcohol regularly? | □ yes | □ no |
| Do you suffer from a mental disorder? | □ yes | □ no |
| Are you pregnant? | □ yes | □ no |
| Do you show self-harm behavior? | □ yes | □ no |
| Do you think about suicide? * | □ yes | □ no |
| ** If yes:*   \| Have you tried to commit suicide in the past? \| □ yes \| □ no \| \| --- \| --- \| --- \| \| Do you have plans or ideas how to commit suicide? \| □ yes \| □ no \| \| Have you made preparations? \| □ yes \| □ no \| \| Has someone out of your family or friends committed suicide in the past? \| □ yes \| □ no \|  - Arrangement of counseling with the supervisor (clinical psychologist) | | |
| *If NOT all inclusion criteria are matched:* “Thank you very much for your disclosure. Unfortunately, you can’t participate in the study because of our inclusion and exclusion criteria. Your personal information will be deleted after our telephone interview. I wish you all the best, thank you for your willingness to participate. Goodbye.” | | |
| *If all inclusion criteria are matched:* “Thank you very much for your disclosure. You are eligible to participate in our study. I would like to set an appointment for the first session with you now. What about (date/time)?” | | |

**Standardized telephone screening**
